# Mission valence of civil servants: development and validation of a three-dimensional scale

**DOI:** 10.3389/fpsyg.2025.1734019

**Published:** 2026-01-12

**Authors:** Xi Liu, Mengchu Zhao, Weibo Zheng

**Affiliations:** 1 College of Public Administration, Huazhong University of Science and Technology, Wuhan, China; 2 School of Law and Humanities, Zhejiang Sci-Tech University, Hangzhou, China

**Keywords:** civil servants' mission valence, dimension exploration, grounded theory, public sector, scale development

## Abstract

**Background:**

In the context of public service reform and sustained governance optimization, mission valence is a pivotal driver of public service behavior, yet existing measurements are predominantly unidimensional. These simplified tools often fail to capture the complex psychological process of civil servants within the organizational settings of the public sector. A multidimensional instrument is therefore essential to accurately reflect this construct.

**Methods and results:**

This study develops and validates a multidimensional mission valence scale for civil servants using a mixed-methods design. Study 1 (*N* = 21) conducted grounded theory interviews to derive content domains and initial items inductively. Study 2 firstly applied item analysis and exploratory factor analysis (*N* = 205), yielding a 12-item scale with three dimensions: Value Attribute Assessment, Affective Role Identity, and Behavioral Transformation Intention. Subsequently, we conducted confirmatory factor analysis (*N* = 216) to verify the structure and assess reliability and validity. Across samples, the model fit was good, internal consistency and composite reliability were strong, and convergent and discriminant validity were satisfactory. Study 3 (*N* = 456) supported criterion-related validity, indicating that servant leadership exhibits a significant positive association with mission valence, mediated by public service motivation.

**Conclusion:**

The findings developed a psychometrically sound instrument that reconstructs civil servants’ mission valence as a multidimensional process. This validated scale provides a precise tool for examining the mechanisms underlying civil servants’ mission valence in public sector organizational behavior.

## 1 Introduction

Amidst the imperatives of sustained public sector reform and service delivery optimization, identifying effective motivational mechanisms for civil servants stands as a pivotal concern for elevating administrative efficiency and public governance (Moon and Lim, 2025). While traditional material incentives (e.g., compensation, promotion) retain an important role in organizational management, extant research indicates that exclusive reliance on extrinsic motivators often fails to yield enduring and profound effects within the public sector (Park, 2022). This is primarily due to strict budgetary constraints and the inherent multiplicity and ambiguity of public organizational goals. In contrast, mission valence is considered a core psychological factor driving proactive behavior among public employees. Distinct from general private sector employees, the specific cohort focused on in this study—civil servants—functions as knowledge workers who exercise public power and manage public affairs. They are often characterized by a stronger desire for self-actualization and a profound public service ethos (Venketsamy and Lew, 2022). Thus, for this critical cohort, the organizational mission is not merely a job directive but a fundamental source of their professional meaning.

It is crucial to recognize that mission valence is not a context-free concept. Rather, its perception is deeply embedded in specific institutional and cultural contexts. First, China’s traditional cultural values are highly influential. These values, which historically emphasize “Family-State Sentiment” and “The World is for All,” compel Chinese civil servants to link the organizational mission directly with the grand narratives of national rejuvenation and revitalization. Furthermore, the modern state governance system imposes distinct political commitments. Chinese civil servants bear the political ethic and moral code of “serving the people,” which transcends mere professional requirements. More importantly, China’s distinctive governance style (i.e., emphasizing dedication and collectivism) and the “Red Gene” formed during the modern history of national salvation and reconstruction collectively shape the unique psychological and cognitive patterns of the Chinese civil service. This distinctive cultural and institutional environment is why indigenous research is essential. It provides the necessary foundation for the bottom-up, empirically grounded approach used in this study. Applying measurement instruments developed in Western cultural contexts may fail to accurately capture the authentic perceptions and complex experiences of civil servants toward their organizational mission. Therefore, the Chinese public sector context provides a uniquely fitting research setting for the operationalization of this specialized construct.

Based on the aforementioned unique group characteristics and the specific research context, this study posits that the connotation and extension of civil servants’ mission valence are significantly richer than the initial definition proposed by Rainey and Steinbauer (1999). More precisely, it offers a focused explanation of the psychological process through which civil servants transform macro-level organizational goals into specific, personal work motivation within their organizational setting. Therefore, it is crucial to clearly distinguish the construct of civil servants’ mission valence from similar concepts to mitigate the risk of conceptual overlap. For instance, it is fundamentally not an individual trait like public service motivation (Perry and Wise, 1990). Rather, it is a situational perception and evaluative psychological process. Similarly, it is neither a notion of destiny or calling that transcends organizational boundaries (Dik and Duffy, 2009), nor is it a meaning-making process derived from a broad or diffuse source (Ashforth and Mael, 1989; Rosso et al., 2010). Instead, civil servants’ mission valence is precisely defined as a psychological state that is specifically sourced from the organizational mission and directly targeted toward specific organizational goals. This explicit contextual and source-based boundary ensures the theoretical distinctiveness of the construct, thereby underscoring the necessity of thoroughly investigating the conceptual extension and structural dimensions of civil servants’ mission valence.

Existing research has predominantly concentrated on exploring the impact of mission valence through simple manifestations, such as job importance (Wright, 2007) or emotional arousal (Bosak et al., 2021). Thus, studies often utilize unidimensional scales. However, this expedient approach risks overlooking the rich, multi-faceted nature of mission valence as a complex psychological process. This limitation constrains a deep understanding of its underlying mechanisms. Therefore, this study aims to root research within the specific context of the Chinese public sector. To achieve this, we employ an inductive approach. This discovery-oriented path allows us to delve into the genuine feelings and experiences of Chinese civil servants inspired by their organizational mission. Instead of relying on pre-existing theoretical assumptions to create an operational concept, this methodology aims to explore and refine the structural dimensions of mission valence from the ground up. By doing so, we aim to break through the constraints of current unidimensional measures.

To this end, the overarching objective of this study is to develop and validate a reliable, multidimensional scale for civil servants’ mission valence. This instrument will serve a dual purpose. First, it provides a precise measurement tool to explain the behavior of Chinese civil servants from a psychological perspective. Second, it lays a solid foundation for future empirical examinations of the formation mechanisms, effects, and boundary conditions of mission valence. Ultimately, this research makes a nuanced theoretical contribution to the fields of public sector organizational behavior and mission-based motivation theory.

## 2 Literature review

### 2.1 The conceptual connotation of mission valence

The term ‘mission valence’ was first introduced by Rainey and Steinbauer in their discussion of effective government organization theory (Rainey and Steinbauer, 1999), drawing on the concept of ‘valence’ from Vroom’s Expectancy Theory (Vroom, 1964). Expectancy theory states that ‘valence’ refers to the degree of preference or the value assigned by an individual to a specific outcome (Vroom, 1964). Based on this, mission valence is used to describe the degree of employee engagement, attraction, and perception of the value of the organization’s mission (Wright, 2007). It not only reflects an individual’s understanding of the organization’s values but also indicates the level of employee integration with the organization’s mission.

As research deepens, scholars have explored the connotations of mission valence from different perspectives, which can primarily be divided into the individual perception perspective and the organizational effectiveness perspective. From the individual perception perspective, mission valence is defined as the importance and attraction of the organizational mission as perceived by the employee (Wright et al., 2012). When employees are attracted by the vision or mission values of their organization, they experience strong intrinsic motivation and exhibit more proactive work behaviors (Liu et al., 2024). In other words, mission valence is an important factor driving employee motivation and behavior. At the same time, it also reflects the degree of internal consistency between an individual’s values and the long-term goals pursued by the organization (Pandey et al., 2008).

From the organizational effectiveness perspective, mission valence emphasizes the significant importance of an individual’s contribution to achieving the organization’s task goals (Willems et al., 2021). A clear and compelling organizational mission can show employees how their daily work contributes to achieving organizational goals and benefiting society, thereby guiding them to complete tasks efficiently (Vogel et al., 2023). An organizational mission that is valued and attractive is more likely to gain the support of societal individuals and continuously motivate organizational members to display more proactive behaviors (Caillier, 2015). Therefore, mission valence is of significant importance for aspects such as employee recruitment, team stability, and behavioral motivation within an organization.

Based on existing research, scholars have different entry points for defining mission valence, and there is a lack of clarity and unity, with no consistent theoretical perspective. Existing literature, when exploring its concept, either focuses on individual perception or organizational effectiveness, and has yet to form an integrated definitional framework.

Caillier (2014) argues that mission valence is not merely a cognition of job importance; rather, it reflects, at a deeper level, the extent to which the organizational mission is internalized psychologically by the individual. Therefore, drawing on an integrated perspective of Person-Organization Fit Theory and Expectancy Theory, this study posits that these two facets of mission valence constitute a unified cognitive process rather than functioning as merely juxtaposed or opposing categories.

Specifically, mission valence embodies the transformation from the organization’s objective attributes to the individual’s subjective orientation:

First, Organizational Effectiveness as Antecedent. From the perspective of organizational effectiveness, the mission embodies the social value and significance of organizational goals (Willems et al., 2021). A clear and socially impactful mission serves as a signal. It demonstrates to public servants how the public sector exerts a positive influence on society through daily operations. This establishes the objective foundation of mission valence.

Second, Individual Perception as Internalization. Merely possessing a noble mission is insufficient; civil servants must perceive and process this value psychologically. When the values of individual public servants resonate with the organization’s social goals—creating a state of value congruence—the organizational mission transforms into a personal appeal for the employee (Wright et al., 2012).

In summary, the objective value of the mission at the organizational level serves as the basis for individual perception. Conversely, this individual perception constitutes the essential pathway through which the organizational mission achieves its effectiveness.

Therefore, within this integrated theoretical framework, this study argues that mission valence should not be conceptualized merely as the extent of an individual’s “cognition of job importance” or “emotional arousal.” Instead, it should be viewed as a comprehensive psychological state grounded in person-organization value fit.

This state embodies the degree to which civil servants internalize the social function of the organization (from the organizational perspective) into the intrinsic meaning of their own work (from the individual perspective).

Consequently, the varying levels of mission valence among employees within the same organization stem from individual differences in the assessment and internalization of the organization’s objective mission.

### 2.2 Measurement methods and tools for mission valence

Currently, research on the measurement of mission valence is still in its early stages, and the measurement tools used are relatively limited. Existing research primarily measures mission valence using Likert scales, with employees self-assessing their perception of mission valence. Based on their focus, these measurement tools can generally be classified into two categories:

The first category focuses on measuring employees’ perception of the importance of the organization’s work. Among them, the most representative is the unidimensional scale developed by Wright (2007), which includes three items. A typical item is: ‘This division provides valuable public services.’ Due to its simplicity and universality, this scale has become one of the most widely used measurement tools in subsequent research.

For instance, Wright et al. (2012) applied an adapted version of this instrument to a survey of 1,538 local government department heads in the United States, reporting a Cronbach’s *α* of 0.84, which indicates good internal consistency.

Subsequently, Guerrero and Chênevert (2021) employed the scale in a large-sample study of 2,576 Canadian municipal employees. Their confirmatory factor analysis supported the single-factor structure, and the scale demonstrated a high reliability coefficient of 0.90, further validating the instrument’s stability within the North American public sector context.

The second category focuses on measuring the extent to which the mission stimulates an individual’s emotional arousal. For example, the scale developed by Bosak et al. (2021) focuses on the motivational and inspirational effects of the organizational mission on employees. A typical item is: ‘I am inspired by the organizational mission’. In a study involving 185 employees within the Canadian hospital system, they validated the scale using a time-lagged design. The results indicated high internal consistency (Cronbach’s *α* = 0.92). This finding demonstrates that the instrument possesses robust measurement properties within the healthcare sector—a profession characterized by a strong sense of mission.

Additionally, Pandey et al. (2008) expanded upon Wright’s (2007) scale by adding the item ‘For me, the mission of this organization is exciting,’ creating a four-item scale that combines the perception of work importance with individual emotional arousal. This scale has also been widely adopted by subsequent researchers.

For example, Wright and Pandey (2011) employed an adapted version of this scale in a study of 284 senior executives in U.S. local governments. They reported a Cronbach’s *α* of 0.85, indicating good internal consistency.

Similarly, Pandey et al. (2008) utilized the scale in a survey of 1,276 managers across state health and human service agencies in the United States. Their study reported a reliability coefficient of 0.84, with factor loadings all exceeding 0.60, suggesting that the scale effectively captures public managers’ emotional commitment to the mission.

Furthermore, Caillier (2016) applied this affect-inclusive scale in a study involving 778 employees from federal, state, and local governments in the U.S. The study yielded a Cronbach’s *α* of 0.78, further verifying the scale’s applicability across different levels of government.

Finally, Willems et al. (2021) used the scale to measure subjects’ reactions to organizational mission in two experimental studies regarding organizational reputation recovery, with sample sizes of 304 and 582, respectively. They reported a Cronbach’s *α* reaching 0.93, confirming the reliability of the instrument in capturing mission perception within experimental contexts.

It is evident that existing research has made some progress in exploring the structure of mission valence and its measurement tools, but there are still certain limitations. Firstly, existing measurement tools primarily exhibit a unidimensional characteristic, making it difficult to comprehensively reflect mission valence as a complex psychological construct. Existing research mainly focuses on exploring its expression in singular aspects such as ‘job importance’ or ‘emotional arousal.’ While this approach is convenient, it may overlook the rich connotations of mission valence as a complex psychological process, thereby limiting a deeper understanding of its underlying mechanisms.

Secondly, although studies applying the scales developed by Wright (2007) and Pandey et al. (2008) have demonstrated acceptable internal consistency and structural validity through factor analysis in specific samples, the item generation process for these instruments primarily relied on theoretical deduction or adaptations from private sector scales.

While this “top-down” approach ensures statistical reliability and validity, it may fail to fully capture the rich content and authentic experiences of civil servants regarding mission perception during the construction phase. Consequently, these measures may suffer from potential deficiencies in content validity.

Finally, most of these scales originate from specific cultural contexts, and the few empirical studies generally cite them directly. Some studies, in the interest of convenience, select only certain items for measurement, raising concerns about their cross-cultural adaptability and content validity. These measurement limitations have not only hindered empirical testing of the antecedents and consequences of mission valence to some extent, but also created a bottleneck in the theoretical development of this construct.

In view of the limitations of existing research, this study is grounded in the authentic work experiences of civil servants and adopts a “bottom-up” grounded theory approach to systematically investigate mission valence.

Charmaz (2006) suggests that this method may face potential threats such as researcher bias and narrow generalizability. Therefore, this study employs a mixed-methods design.

Specifically, first, based on the grounded theory method, this study deeply explores the conceptual connotations of mission valence and preliminarily constructs its multidimensional theoretical framework to enrich and expand the concept of mission valence. Secondly, a measurement scale for mission valence is developed, and through exploratory factor analysis and confirmatory factor analysis, the objectivity and generalizability of quantitative data are leveraged to compensate, to some extent, for the potential subjectivity and limitations inherent in qualitative methods, the scale is ensured to have good reliability, validity, and applicability, accurately reflecting the conceptual meaning of mission valence, and providing an operationalizable measurement tool for subsequent empirical research. Finally, based on the developed measurement tool, the study preliminarily validates the impact of servant leadership on mission valence and other key variables, to test the criterion-related validity of the scale and further explore the theoretical and practical research value of this construct.

## 3 Study 1: Constructing the dimensions of mission valence based on grounded theory

Grounded theory is a qualitative research method that requires researchers to inductively derive empirical generalizations from direct observations or raw data, and then elevate them to systematic theories (Charmaz and Thornberg, 2021). Since current research on mission valence mostly remains at the conceptual discussion stage and lacks systematic dimensional analysis, pure theoretical deduction is insufficient to comprehensively reveal its rich connotations. Therefore, based on the grounded theory approach, this study will adopt the procedural analysis framework of Corbin and Strauss (1990), using steps such as theoretical sampling, data collection, data coding, and concept refinement to construct a multidimensional construct of mission valence for civil servants, thus addressing the limitations of the existing unidimensional perspective.

### 3.1 Theoretical sampling

Theoretical sampling is the purposeful selection of samples based on the needs of the construct theory (Glaser, 1978). In this study, mission valence is a key psychological cognition formed by civil servants under the influence of the organization’s mission. Its connotation and formation process are deeply influenced by individual work situations and the organizational environment. Therefore, in order to comprehensively and deeply explore the dimensional construct of mission valence, this study employed a purposive sampling strategy, aiming to select typical cases that could provide rich information.

Specifically, to ensure the diversity and representativeness of the sample, this study constructed a multidimensional sampling framework, aiming to cover public sector employee groups with different characteristics. Firstly, in terms of regional selection, to reflect organizational contexts under different levels of economic and social development, the sample covers multiple regions from Eastern, Central, and Western China. Secondly, in terms of organizational types, the sample includes various public sector departments, such as the Development and Reform Commission, Civil Affairs Bureau, Human Resources and Social Security Bureau, and Health Commission, to capture the differentiated experiences brought about by different organizational missions. Finally, in terms of hierarchical distribution, the sample includes civil servants from both urban and township levels, ensuring the generalizability of the research findings.

This study determined the final sample size based on the principle of theoretical saturation (Strauss and Corbin, 1997). Following the Constant Comparative Method, we adopted an iterative strategy of simultaneous data collection and analysis. Specifically, the core categories stabilized after the analysis of the 18th interview, with no new significant codes emerging. The subsequent three interviews (i.e., the 19th, 20th, and 21st participants) served as confirmatory cases; as these interviews did not reveal new theoretical dimensions, we determined that saturation had been reached and terminated sampling, resulting in a final sample size of 21.

The sample exhibits good heterogeneity in terms of gender, age, educational background, and years of work experience, providing a foundation for the subsequent inductive extraction and refinement of theoretical constructs from the raw data.

### 3.2 Data collection

This study primarily uses semi-structured in-depth interviews to collect data. A semi-structured interview follows a general interview guide, allowing the researcher to flexibly adjust the questioning approach and order based on the actual situation during the conversation, to deeply explore the interviewees’ authentic experiences and underlying cognitions. Before conducting the interviews, to ensure the scientific and practical relevance of the interview guide, this study first formed an expert advisory team consisting of experts in public sector human resource management, relevant doctoral students, and frontline civil servants with rich practical experience. This team conducted multiple rounds of review and revision of the initial interview guide. Subsequently, the interview guide was tested through pilot interviews, and the final standardized interview guide was confirmed, including four core questions: ‘What is the mission of your organization? Please briefly explain it based on your understanding,’ ‘How do you view the mission of your organization, and what value do you think it holds?’ ‘How do you think the mission of your organization is related to the specific work you do? Please share your understanding,’ and ‘What impact do you think the mission of your organization has on you? Please discuss with specific examples.’

After finalizing the interview guide, this study involved 21 civil servants in semi-structured, one-on-one depth interviews conducted via both face-to-face meetings and telephone calls, with each session lasting between 30 and 60 min. Our primary goal was to comprehensively capture how employees perceive, value, and are influenced by their organizational mission. To facilitate open dialogue, we typically began by asking participants to describe their general work experiences to help them feel at ease, and then encouraged them to provide specific examples while using probing questions to deepen our understanding of their inner thoughts regarding the mission. All interviews were recorded with informed consent and fully transcribed, yielding over 120,000 words of raw data strictly reserved for academic analysis.

All research in this paper was conducted in accordance with the Declaration of Helsinki. Ethical review and approval were waived for this study, as per Article 32 of the Interpretation of the Ethical Review Measures for Life Sciences and Medical Research Involving Human Beings in China. All interviews were recorded after obtaining informed consent from all subjects involved in the study. All audio files were transcribed into text, resulting in over 120,000 words of raw interview data.

To ensure the objectivity and reliability of the subsequent coding, this study strictly adhered to an inter-rater reliability check procedure. Specifically, two trained research team members first independently coded 5 randomly selected interview transcripts (accounting for approximately 25% of the total sample).

The calculated Cohen’s Kappa coefficient was 0.853, indicating a good level of agreement between the coders (Landis and Koch, 1977). Upon confirming that the reliability met the required standards, the two researchers proceeded to code the remaining data.

Regarding sporadic discrepancies that arose during the coding process, we invited a professor with extensive experience in qualitative research to provide guidance. Consensus was reached through anonymous group discussions, thereby mitigating the risk of post-hoc subjective convergence to a certain extent.

### 3.3 Ethics statement

Although formal ethics board approval was not mandatory according to institutional and national regulations, as the present study involved neither human clinical trials nor animal experiments. We nevertheless implemented enhanced ethical protocols given the unique professional sensitivities of the Chinese civil service. The research was conducted in strict adherence to the Declaration of Helsinki. All respondents were fully informed of the academic purpose of the study and provided written informed consent. Furthermore, considering the strict hierarchical and political context of the public sector, we enforced rigorous confidentiality and anonymity measures to ensure participant safety and data privacy.

### 3.4 Coding process

#### 3.4.1 Open coding

The core task of open coding is to conceptualize and categorize the initially obtained interview data. Researchers are required to stay close to the raw data and extract initial concepts that reflect the research phenomena. At this stage, this study conducted a detailed line-by-line and sentence-by-sentence analysis of the 21 interview transcripts. During the coding process, this study maintained an open research attitude, striving to objectively and authentically present the interviewees’ perceptions and experiences of the organization’s mission, extracting key statements and core terms from the interviews to form initial concepts. This process aims to allow the concepts inherent in the data to naturally emerge based on the characteristics of the data itself, ensuring that each coding label accurately reflects its core meaning. Finally, through a systematic analysis of all the interview data, this study identified and extracted 132 initial concepts related to mission valence. To visually demonstrate the coding process, some examples are shown in Table 1.

**Table 1:** Open coding (example).

Interview coding	Original material	Initial concept
C3-2	We primarily manage the cultural and sports center and cultural square in the town, regularly organizing activities for the villagers, such as square dance competitions and weekend classes for left-behind children. These resources make rural life more enriching.	Public resource management
G2-5	By maintaining market order and improving the business environment, we have promoted local economic development. For example, last year, many small and micro enterprises were established, driving regional employment.	Regional economic prosperity
S5-7	We have built standardized health clinics in remote areas and purchased a lot of basic medical equipment, allowing villagers to access nearby medical services and solving the problem of difficult access to healthcare.	Healthcare resource optimization
H1-3	Whenever I think about our organization’s mission to promote economic development, I feel a great sense of responsibility, which motivates me to work even harder.	Mission appeal
G1-3	The mission of serving the people makes me feel that I am doing something very meaningful, which gives me great motivation.	Altruism
S3-4	Our organization emphasizes teamwork, and everyone works towards the common mission, which makes me feel that my work is meaningful.	Team collaboration
C4-7	The organization’s mission helps me maintain a positive attitude at work, and even when facing difficulties, I can approach them with optimism.	Positive emotions
H3-6	Thinking about the organization’s mission, I feel energized every day. Even when the work is exhausting, I still try my best to do it.	Energetic
C2-2	I pay attention to the details of my daily work because I know it directly impacts the service quality of the organization and whether the public approves of it.	Work quality
H2-5	I am very willing to help community residents, such as organizing volunteer activities and providing consultation services.	Voluntary service

To facilitate the identification of interviewee information sources, civil servants from Guangdong Province are marked with ‘G’ (e.g., G1, G2, .), those from Hubei Province with ‘H’ (e.g., H1, H2, .), those from Shaanxi Province with ‘S’ (e.g., S1, S2, .), and those from Sichuan Province with ‘C’ (e.g., C1, C2, .). For example, ‘G1-1’ refers to the first key point mentioned by the first civil servant from Guangdong in the interview, and similarly for the rest of the text.

#### 3.4.2 Axial coding

Based on the initial concepts obtained from open coding, this study entered the axial coding stages, aiming to cluster and refine the concepts, uncover their underlying structure, and form more generalized core categories. This study repeatedly compared, selected, and synthesized the 132 initial concepts extracted during the open coding process. Initially, 24 subcategories were identified, and these were further integrated into 12 main categories, such as ‘publicness,’ ‘emotional resonance,’ ‘role recognition,’ and ‘behavioral energy’ (see Table 2). The entire inductive process follows a logic progressing from the concrete to the abstract. The specific derivation process is illustrated as follows:

**Table 2:** Example of axial coding analysis.

Initial concept	Category	Main category
G1-2 Social infrastructure construction; …	Types of public services	Publicness strategic nature
C2-5 Improve social equity; …	Impact of public services	
C4-1 Economic revitalization; …	National development goals	Regionality inclusiveness
H2-3 Enhance international competitiveness; …	Contribution to national strategy	
G2-5 Regional economic prosperity; …	Regional development	Self-interest emotional resonance
H4-1 Social harmony; …	Social progress	
S1-6 Improved living standards; …	Improvement of people’s livelihood	External motivation role recognition
S3-2 Enhance social welfare; …	Social security	
C3-8 Professional skill enhancement; …	Career growth	Emotional Intensity Behavioral energy
G1-5 Realize life’s value; …	Self-actualization	
H1-3 Mission appeal; …	Spiritual motivation	Behavioral intention
H3-5 Sense of pride; …	Emotional resonance	
G1-3 Altruism; …	Service motivation	Publicness strategic nature
H1-1 Clear service goals; …	Service identification	
G2-6 Personal responsibilities; …	Role identification	Regionality inclusiveness
G3-3 Influence on society; …	Perception of impact	
H4-6 High enthusiasm; …	Emotional experience	Self-interest emotional resonance
G2-4 Improve work efficiency; …	Action motivation	
H3-6 Energetic; …	Work engagement	External motivation role recognition
S1-7 Proactive; …	Work attitude	
G2-2 Task completion; …	Work Focus	Emotional intensity Behavioral energy
S4-5 Long-term persistence; …	Work continuity	
H2-5 Voluntary service; …	Willingness to serve	Behavioral intention
C1-6 Service satisfaction; …	Service experience	

Source: Compiled by the authors.

First, regarding the dimension of Value Attribute Assessment, this study focuses on public servants’ evaluations of the objective attributes of the organizational mission.

For instance, during interviews, respondents mentioned phrases such as “maintaining public security in the jurisdiction,” “eliminating safety hazards,” and “guaranteeing the stable supply of water, electricity, and gas.” These raw data were first conceptualized into initial concepts such as “maintenance of social order” and “infrastructure guarantee.” Since these concepts collectively point to the organization’s foundational functions in maintaining social operations, they were categorized into the sub-categories of Basic Livelihood Guarantee and Social Order Maintenance, which were eventually aggregated into the main category of Publicness.

Similarly, when respondents mentioned “implementing digital government construction,” “promoting institutional reform,” and “participating in key national projects,” these concepts were identified as possessing macro-level significance beyond specific tasks. Consequently, they were classified into the sub-categories of National Strategy Implementation and Reform and Innovation Drive, which were further refined into the main category of Strategic Nature.

Following this logic, the study also identified other main categories, including Regionality (comprising regional economic promotion and inheritance of local characteristics), Inclusiveness (comprising improvement of people’s wellbeing and social fairness and justice), and Self-Interest (comprising a career growth platform). Together, these categories constitute the multifaceted judgment of mission value by public servants.

Second, regarding the dimension of Affective Role Identity, the analysis focuses on the process by which the mission is internalized into individual emotions and identity.

For example, Respondent G1 stated, “The mission of serving the people makes me feel that I am doing something very meaningful,” while Respondent H1 noted, “Every time I think about our unit’s mission, I feel a great weight of responsibility on my shoulders.” These statements were coded into initial concepts such as “mission appeal,” “value identification,” and “personal duty.”

Among these, concepts involving emotional contagion and value resonance (e.g., “feeling inspired”) were categorized into sub-categories such as Sense of Value Identification, which were further abstracted into the main category of Affective Resonance.

Conversely, concepts involving responsibility confirmation and identity positioning (e.g., “daring not to slack off”) were classified into sub-categories such as Responsibility Awareness. These were ultimately aggregated into the main category of Role Cognition.

In addition, positive feedback from external sources (e.g., “gratitude from the public”) was summarized into the main category of External Motivation.

Finally, regarding the dimension of Behavioral Transformation Intention, this study examines the energy states and action tendencies triggered by the mission.

The analysis revealed that respondents frequently used expressions such as “full of drive,” “willing to work even if tired,” and “actively thinking about optimizing processes.” These descriptions were distilled into initial concepts such as “energetic,” “time investment,” and “active improvement.”

By analyzing their intrinsic attributes, concepts describing psychological energy states (e.g., “high enthusiasm”) were categorized into the main category of Affective Intensity, while concepts describing the readiness for physical and time investment (e.g., “vitality stimulation”) were categorized into the main category of Behavioral Energy.

Meanwhile, intentions directed toward work performance improvement (e.g., “pursuing work standards”) were categorized into the main category of Behavioral Intention, and intentions directed toward extra-role activities (e.g., “volunteer service”) were categorized into the main category of Altruistic Behavior Drive.

#### 3.4.3 Selective coding

Selective coding aims to integrate all main categories by identifying core categories, thereby constructing a logically rigorous theoretical framework.

Through iterative comparison and abstraction of the 12 main categories, this study identified intrinsic differences regarding their conceptual nature and logical orientation. Consequently, three core categories were derived (Table 3):

**Table 3:** Selective coding results.

Main category	Main category
Publicness strategic nature	Value attribute assessment
Regionality inclusiveness	
Self-interest emotional resonance	
External motivation role recognition	
Emotional intensity behavioral energy	
Behavioral intention	Affective role identity
Publicness strategic nature	
Regionality inclusiveness	
Self-interest emotional resonance	
External motivation role recognition	Behavioral transformation intention
Emotional intensity behavioral energy	
Behavioral intention	

Source: Compiled by the authors.

First, Value Attribute Assessment. This core category integrates five main categories: Publicness, Strategic Nature, Regionality, Inclusiveness, and Self-Interest.

The commonality among these categories lies in their focus on the attributes of the “organizational mission” as an object across different levels. They encompass macro-level national strategic significance (e.g., “national rejuvenation”), meso-level regional social contribution (e.g., “local development”), and micro-level individual instrumental value (e.g., “personal growth”).

Studies indicate that public servants’ perception of the mission initiates with the rational identification and evaluation of these objective attributes. Therefore, regardless of whether the attributes are directed toward the external society or the internal self, they essentially represent the individual’s comprehensive cognitive judgment of the mission’s value. Consequently, this study unifies these evaluations regarding the objective attributes of the organizational mission under the category of Value Attribute Assessment.

This construct is defined as the employee’s subjective evaluation of the comprehensive value possessed by the organizational mission across multiple levels—including the nation, society, the public, and the individual. It reflects the employee’s cognitive linkage of the organizational mission from macro-level strategies to micro-level contributions.

Second, Affective Role Identity. This core category aggregates the main categories of Affective Resonance, Role Cognition, and External Motivation.

Unlike the previous category, which focused on the “object,” these categories shift focus to the “relationship between the subject (employee) and the object (mission).” Specifically, once employees complete the cognitive assessment of the mission’s value, they generate an internalization response.

Within this context, Affective Resonance reflects the emotional experience of the individual after being touched by the mission, while Role Cognition reflects the identity positioning where the individual incorporates the self into the mission framework.

This process marks a transition in mission perception from rational evaluation to internal connection, embodying a deep integration of the individual and the organizational mission in terms of emotion and identity. Therefore, it is categorized as Affective Role Identity.

This study defines this category as the emotional resonance employees develop toward the organizational mission, the clear positioning of their own roles within that mission, and the internal motivation felt as a result. It reflects the psychological tendency of the individual progressing from being attracted by the mission to establishing self-positioning.

Third, Behavioral Transformation Intention. This core category encompasses the three main categories of Behavioral Energy, Behavioral Intention, and Altruistic Behavior Drive.

This core category represents the final output of the preceding cognitive and affective psychological processes. Interview data indicate that high levels of mission valence eventually externalize into a state where employees are “ready to act,” manifested as a surge in work vitality and a tendency toward proactive service.

Therefore, this category encapsulates the intentionality with which mission perception drives the individual to transition from a psychological state to actual action; thus, it is summarized as Behavioral Transformation Intention.

This study defines this category as the degree of employees’ willingness to transform their perception of and identification with the mission into concrete work engagement and service behaviors. It reflects the driving effect of mission perception on employees’ work vitality, proactive engagement, and altruistic behavioral intentions.

## 4 Study 2: Development and validation of the mission valence scale

### 4.1 Scale development

Based on the mission valence construct developed through grounded theory analysis and incorporating the data coding from the in-depth interviews, this study initially formulated 24 descriptive statements with relatively clear semantics to measure the three dimensions of ‘Value Attribute Assessment,’ ‘Affective Role Identity,’ and ‘Behavioral Transformation Intention.’

To ensure the content validity of the scale, this study first convened an expert panel comprising nine members to evaluate the item pool.

Specifically, four professors specializing in public sector human resource management were invited as theoretical experts, alongside five incumbent public servants with over 10 years of work experience acting as practical experts. They scrutinized the basic content, wording, and dimensional affiliation of the initial items to ensure that the items were semantically clear and concise, and closely aligned with the core dimensions of the theoretical construct.

After integrating the experts’ feedback, the items were refined to 20 through revisions, consolidations, and deletions of terms. Subsequently, to further assess the readability and accuracy of the scale within the target group, this study invited another group of frontline civil servants to evaluate the questionnaire.

Based on their feedback, the research team revised and consolidated the scale, further refining it to 17 measurement items.

Next, this study invited members of the expert panel to independently rate all items using a 4-point scale (1 = irrelevant, 4 = highly relevant). Based on the rating results, the content validity index was calculated.

The results indicated that the Item-Level Content Validity Index (I-CVI) for the retained items consistently exceeded 0.78, and the Scale-Level Content Validity Index (S-CVI/Ave) reached 0.955, demonstrating that the scale possesses good content validity (Polit and Beck, 2006).

In summary, an initial scale comprising 17 items was finally established.

### 4.2 Exploratory factor analysis

This study employed Exploratory Factor Analysis to examine the factor structure constructed for measuring the core constructs, thereby clarifying the latent structure and dimensions of the mission valence scale.

#### 4.2.1 Sampling and measurement

The survey was conducted through an online questionnaire platform, distributing questionnaires to civil servants from various provincial and government departments at different levels. A total of 240 questionnaires were distributed, and after excluding those with missing, incomplete, or abnormally timed responses, 205 valid samples were obtained, resulting in a valid response rate of 85.41%. The sample characteristics are as follows: 66.34% of the respondents are male; 60.98% have a master’s degree or higher; 68.78% are aged 35 or older.

It is worth noting that the educational level and age distribution of this sample are slightly higher than the average for the general grassroots civil servant population. This may be attributed to the fact that the data collection channels were primarily concentrated in provincial and municipal agencies, and the respondents were mainly key operational staff.

Although this sample characteristic may, to a certain extent, limit the generalizability of the results to civil servants with lower educational backgrounds or those in junior positions, during the exploratory stage of scale development, participants with higher education levels typically possess a more accurate capability to comprehend the relatively abstract psychological construct of “mission valence.” This contributes to ensuring the accuracy of item comprehension and the clarity of the factor structure.

This issue will be further discussed in the limitations section. In addition, the survey questionnaire used the initial mission valence scale with 17 items developed earlier, and the measurement was conducted using a 5-point Likert scale (1 = ‘Strongly Disagree’, 5 = ‘Strongly Agree’).

#### 4.2.2 Analysis results

Before conducting the exploratory factor analysis, this study first employed Cronbach’s *α* coefficient to test the reliability of the questionnaire and assess its internal consistency. According to previous research, when used for exploratory analysis, an item demonstrates good reliability and should be retained if the Cronbach’s α of its corresponding factor exceeds 0.6 and the corrected item-total correlation (CITC) of the item is greater than 0.5 (Nunnally, 1978). In this study, the initial 17 items were subjected to such a reliability test. The results showed that the CITC values of the following three items—” The mission of our organization is very important for the implementation of its strategy,” “Practicing the mission of our organization gives me a strong sense of accomplishment,” and “The mission of our organization makes me feel responsible for promoting the achievement of organizational goals”—were all below 0.5. Therefore, these items were removed. Subsequently, the suitability of the data for factor analysis was assessed. The Kaiser–Meyer–Olkin (KMO) measure of sampling adequacy for the collected data was 0.916, and Bartlett’s test of sphericity yielded a chi-square value of 3445.751 (*p* < 0.001), indicating that the sample data met the basic prerequisites for factor analysis (Kaiser, 1974).

Subsequently, principal component analysis was conducted, and the remaining 14 items were analyzed using the varimax rotation method to maximize variance. Factor extraction was conducted based on a comprehensive assessment using the criterion of initial eigenvalues greater than 1 and referencing the scree plot results. The results identified three factors with eigenvalues exceeding 1. Simultaneously, observation of the scree plot revealed that the slope of the curve exhibited a break after the third factor and subsequently leveled off. On this basis, items were screened according to criteria requiring factor loadings to exceed 0.5 and the absence of cross-loadings. After multiple rounds of analysis, 12 items were ultimately retained, forming a clear three-factor structure (see Table 4). The factor loadings for each item on the corresponding factors ranged from 0.816 to 0.901, with a cumulative variance explanation of 90.824%, indicating that the factor structure is highly satisfactory.

**Table 4:** Results of exploratory factor analysis (*N* = 205).

Factor name	Measurement items	Factor 1	Factor 2	Factor 3
Value attribute assessment	1. The mission of our organization is to provide valuable public services.	0.881		
	2. The mission of our organization is crucial for the revitalization of the nation.	0.893		
	3. The mission of our organization is beneficial to regional social development.	0.887		
	4. The mission of our organization can enhance the wellbeing of the people.	0.852		
	5. The mission of our organization is beneficial to my personal development.	0.816		
Affective role identity	6. I am inspired by the mission of my organization.		0.873	
	7. The mission of our organization to serve the people motivates me.		0.875	
	8. I know the role I can play in carrying out the mission of my organization.		0.874	
	9. The mission of the organization excites me.		0.852	
Behavioral transformation intention	10. The mission of our organization fills me with energy.			0.891
	11. The mission of our organization makes me want to invest myself in my daily work.			0.865
	12. The mission of our organization makes me eager to serve the people.			0.901
Reliability coefficient		0.959	0.977	0.968
Eigenvalue		7.969	1.812	1.118
Variance explained		35.769	30.471	24.583
Cumulative variance explained		35.769	66.240	90.824

From the results of the exploratory factor analysis, mission valence presents a three-factor structure. Based on the item composition of each factor, they were named as follows: Value Attribute Assessment (5 items), Affective Role Identity (4 items), and Behavioral Transformation Intention (3 items). The new scale with 12 items derived from the exploratory factor analysis has an overall Cronbach’s *α* coefficient of 0.954, and the reliability coefficients of each subscale are above 0.959, indicating that the scale demonstrates good stability and internal consistency.

### 4.3 Confirmatory factor analysis

This study further examines the construct validity of mission valence and the appropriateness of the scale through Confirmatory Factor Analysis. According to academic consensus, Confirmatory Factor Analysis should be conducted using a research sample independent of the one used in Exploratory Factor Analysis (Fokkema and Greiff, 2017).

#### 4.3.1 Sampling and measurement

For this survey, a new sample was selected. Using an online questionnaire platform, 250 questionnaires were distributed to civil servants. After rigorous screening to remove invalid responses, 216 valid questionnaires were obtained, yielding an effective response rate of 86.40%. Among the respondents, 55.09% were male, 66.20% held a master’s degree or higher, and 63.43% were aged over 35. The survey questionnaire used in this study adopted the 12-item mission valence scale revised through exploratory factor analysis. The measurement was conducted using a 5-point Likert scale (1 = “Strongly Disagree,” 5 = “Strongly Agree”).

#### 4.3.2 Analysis results

This study used the maximum likelihood method and conducted confirmatory factor analysis using the structural equation modeling software AMOS 24.0. To further validate the construct validity of Mission Valence, the one-factor three-dimensional model derived from the exploratory factor analysis was used as the baseline model. Additionally, three competing models were constructed for comparison: a one-factor two-dimensional model merging “Value Attribute Assessment” and “Affective Role Identity,” a single-factor model merging all items, and a second-order three-factor model with Mission Valence as a higher-order latent variable.

The fit indices of each model are presented in Table 5. It can be observed that both the one-factor three-dimensional model and the second-order three-factor model exhibit the best fit with the data, and the fit indices of the two models are identical. This phenomenon strongly supports the high consistency in fit between the two models (Gorbatov et al., 2021). The chi-square to degrees of freedom ratio (*χ*^2^/df) is 2.195, which is below the recommended threshold of 3; CFI, NFI, and IFI all exceed 0.9; RMSEA is 0.075, which is below the 0.08 threshold; and SRMR is 0.025, which is below the 0.05 threshold. In contrast, the fit indices for the two-factor and single-factor models were less than ideal and did not meet acceptable levels. Therefore, it can be concluded that the three-factor model is the best-performing model among all the competing models.

**Table 5:** Fit indices for different models (*N* = 216).

Model	*χ* ^2^	df	*χ*^2^/df	GFI	CFI	NFI	RMSEA	SRMR
Second-order three-factor model	111.951	51	2.195	0.918	0.981	0.965	0.075	0.025
Three-factor model	111.951	51	2.195	0.918	0.981	0.965	0.075	0.025
Two-factor model	411.167	53	7.758	0.679	0.887	0.873	0.177	0.052
One-factor model	483.990	54	8.963	0.655	0.864	0.851	0.192	0.055

In addition to the overall model fit, the factor loadings of the measurement items are also a key indicator. In the three-factor model of this study, all standardized factor loadings of the observed variables were above 0.6 and achieved a significance level of *p* < 0.001, indicating that the quality of the measurement items developed in this study is satisfactory.

As indicated by both the previous exploratory factor analysis and theoretical development, the three dimensions of mission valence are moderately to highly correlated. Addressing potential concerns regarding redundancy arising from this high correlation, this study analyzes the issue from two perspectives:

First, as shown in Table 6, the comparison of competing models confirmed that the three-factor structure is significantly superior to the single-factor structure, thereby establishing the discriminant validity of the dimensions.

**Table 6:** Chi-square test of differences (*N* = 216).

Number	Model	*χ* ^2^	df	Model comparison	Δ*χ*^2^	Δdf
1	Three-factor model	111.951	51			
2	Two-factor model	411.167	53	2 vs. 1	299.216***	2
3	One-factor model	483.990	54	3 vs. 1	372.039***	3

***p < 0.001.

Second, this high correlation provides empirical support for the construction of a Second-order Construct. According to Rindskopf and Rose (1988), high correlations among first-order factors imply that they share a higher-order common source of variance.

In this study, the second-order factor of mission valence successfully explained the high covariance among the three first-order factors (standardized path coefficients were all significant and high). This indicates that conceptualizing mission valence as a holistic construct encompassing cognition, affect, and behavioral intention demonstrates greater theoretical parsimony (Principle of Parsimony) and explanatory power than treating them merely as three independently correlated variables (Chen et al., 2005).

Furthermore, since the first-order and second-order three-factor models are statistically equivalent and their fit indices both reached satisfactory levels, this fully confirms that the three-dimensional structure of mission valence proposed in this study possesses robust construct validity. Therefore, this study considers the second-order three-factor model to be the relatively superior model.

#### 4.3.3 Structural validity test results

This study adopts the method proposed by Fornell and Larcker (1981) to determine the convergent validity and discriminant validity of the mission valence scale by combining Composite Reliability (CR) and Average Variance Extracted (AVE).

The research results show (see Table 7) that the Composite Reliability values for the three dimensions of mission valence are 0.958, 0.950, and 0.934, all of which are higher than the 0.70 threshold recommended by Hair et al. (2009), indicating that the three-dimensional structure of this scale has ideal reliability. At the same time, the Average Variance Extracted values for each dimension are 0.821, 0.826, and 0.824, all of which are higher than the 0.5 threshold recommended by Fornell and Larcker (1981), indicating that the scale has good convergent validity.

**Table 7:** Correlation analysis and reliability analysis of dimensions.

Factor	CR	AVE	1	2	3
1. Value attribute assessment	0.958	0.821	(0.906)		
2. Affective role identity	0.950	0.826	0.792	(0.909)	
3. Behavioral transformation intention	0.934	0.824	0.852	0.816	(0.908)

To test the discriminant validity of the scale, this study calculated the square roots of the AVE values for each dimension and compared them with the inter-dimensional correlation coefficients (see Table 7). The results show that the square roots of the AVE values for the three dimensions (the values on the diagonal) are all greater than their corresponding correlation coefficients with the other dimensions. This indicates that the dimensions comprising mission valence are not only correlated but also maintain good discriminant validity, thus forming an integrated whole. In conclusion, the mission valence scale developed in this study demonstrates strong construct validity.

Furthermore, considering the potential issue of insufficient sensitivity associated with this method (Henseler et al., 2015), this study drew upon the work of van der Merr et al. (2024) to conduct a Chi-square difference test by comparing the *χ*^2^ values of the unconstrained measurement model with those of the constrained models. If the *χ*^2^ value of the unconstrained model is significantly lower than that of the constrained model, discriminant validity is established (Zaiţ and Bertea, 2011).

The results indicate (referencing Tables 5, 6) that the *χ*^2^ value for the unconstrained model was 111.951 (df = 51, *χ*^2^/df = 2.195, GFI = 0.918, CFI = 0.981, NFI = 0.965, RMSEA = 0.075). For Constrained Model 1 (two-factor model), the *χ*^2^ value was 411.167 (df = 53, *χ*^2^/df = 7.758, GFI = 0.679, CFI = 0.887, NFI = 0.873, RMSEA = 0.177). For Constrained Model 2 (single-factor model), the *χ*^2^ value was 483.990 (df = 54, *χ*^2^/df = 8.963, GFI = 0.655, CFI = 0.864, NFI = 0.851, RMSEA = 0.192).

The differences in Chi-square values between the two constrained models and the unconstrained model both reached a significance level of 0.001 (△*χ*^2^ = 299.216, *p* < 0.001; △*χ*^2^ = 372.039, *p* < 0.001). These results demonstrate that the Chi-square difference test was significant, indicating good discriminant validity among the dimensions.

In addition, using the competing model comparison method (Anderson and Gerbing, 1988), the indices for the three-factor model were significantly superior to those of the other two models. Synthesizing the analysis above, it is evident that there is robust discriminant validity among the three constructs of mission valence.

## 5 Study 3: The relationship between servant leadership and mission valence of civil servants

In the context of continuous reform and service optimization within the public sector, stimulating civil servants’ intrinsic work motivation and enhancing their identification with organizational goals have become key factors in improving administrative effectiveness (Moon and Lim, 2025). Mission valence, as civil servants’ deep perception of and emotional connection to the value of the organizational mission, is regarded as one of the core psychological factors driving proactive behaviors among civil servants. Therefore, exploring how to effectively cultivate and enhance the mission valence of civil servants has become a focal topic in current public administration research. Previous studies have confirmed that leadership behavior plays a crucial role in shaping the attitudes and motivations of civil servants (Iqbal et al., 2021; Wu and Zhou, 2024). Given that servant leadership emphasizes core features such as concern for subordinates’ growth, employee empowerment, and the cultivation of a service-oriented organizational culture (Xiao et al., 2025), its philosophy aligns closely with enhancing employees’ identification with the public service mission—namely, mission valence. Servant leadership, by meeting employees’ needs, providing supportive resources, and exemplifying altruistic values, is likely to create favorable conditions for employees to perceive and internalize the organizational mission, thereby enhancing their level of mission valence. Therefore, this study posits that servant leadership has a positive effect on civil servants’ mission valence, and this hypothesis will be empirically tested.

### 5.1 Relevant theories and research hypotheses

#### 5.1.1 Servant leadership and mission valence of civil servants

Mission valence refers to the degree of attractiveness and importance that employees perceive in their organization’s mission and its social value (Wright, 2007). It not only reflects an individual’s recognition of organizational values but also indicates the level of psychological internalization of the organization’s mission (Caillier, 2014). Previous research on the factors influencing mission valence has primarily focused on both organizational and individual levels. At the organizational level, leadership style has been identified as a key determinant of employees’ mission valence. Leadership types such as transformational and authentic leadership have been shown to positively enhance employees’ perception of mission valence (Lavigna, 2012; Vogel et al., 2023; Pedersen et al., 2024).

Servant leadership, as a leadership approach that prioritizes serving the needs of subordinates, focuses on the growth and wellbeing of employees, empowers them, and strives to build a harmonious organizational community (Greenleaf, 1977). Based on the Conservation of Resources theory, a leader’s behavior can be regarded as an important organizational resource. Servant leadership, through its distinctive behaviors—such as prioritizing subordinates, supporting their growth and success, providing emotional healing, and empowering through delegation—provides employees with abundant social–emotional and instrumental resources (Najam and Mustamil, 2022). The acquisition of these resources can trigger positive psychological states and behavioral tendencies (Ke et al., 2022).

**Figure 1:**
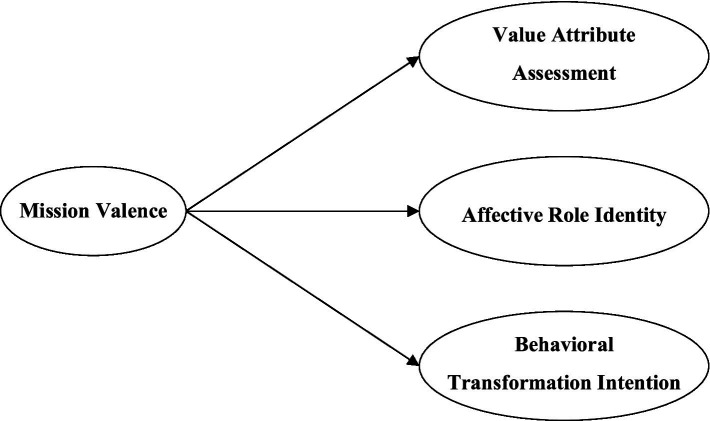
Theoretical conceptualization of mission valence.

Integrating the conceptual connotations of servant leadership and mission valence, this study posits that servant leadership may influence civil servants’ mission valence through the following mechanisms: First, servant leadership emphasizes employees’ personal growth and value realization. By providing development opportunities and support, it helps employees enhance their skills and realize their potential. This enables civil servants to feel more confident and capable in undertaking and fulfilling challenging organizational missions, thereby enhancing their perceived value of the mission (Value Attribute Assessment). Second, servant leadership fosters high-quality trust relationships with employees through genuine care, active listening, and empathy, thereby creating an organizational climate characterized by mutual support and respect. This positive emotional connection and sense of belonging can deepen civil servants’ emotional identification with the organization and its mission (Affective Role Identity). Furthermore, servant leadership emphasizes empowering employees by granting them autonomy and opportunities to participate in decision-making. This not only enhances civil servants’ sense of self-efficacy but also allows them to perceive a strong connection between their work and the organizational mission, thereby stimulating their willingness to translate mission perception into concrete work effort (Behavioral Transformation Intention). Finally, servant leaders typically exhibit a strong moral and service orientation. Their behaviors inherently convey the organization’s public service values, guiding civil servants to focus on the social significance of their work—aligning closely with mission valence, which emphasizes employees’ perception of the organization’s societal contribution (Figure 1).

Based on the discussion above, this study posits that servant leadership—by providing resources, empowering employees, fostering trust, and conveying organizational values—may have a positive effect on civil servants’ mission valence. Therefore, the following hypothesis is proposed:

*H1*: Servant leadership has a significant positive effect on civil servants’ mission valence.

#### 5.1.2 The mediating role of public service motivation

Public Service Motivation, as an intrinsic psychological inclination, belief, and attitude rooted in an individual’s desire to serve society and uphold the public interest, is a key internal factor driving the behavior of civil servants (Liu et al., 2025). Research has shown that public service motivation is significantly positively correlated with employees’ work engagement, organizational commitment, innovative behavior, and task performance (Scrimpshire et al., 2022; Bland et al., 2023; Hussain et al., 2025). According to the active motivation model, public service motivation represents a strong “reason to” drive, compelling employees to exert extra effort to achieve public value. Therefore, civil servants with higher levels of public service motivation are more likely to identify with and be drawn to the organization’s public service mission, thereby exhibiting higher mission valence. Based on this, the study proposes Hypothesis 2a:

*H2a*: Public service motivation has a significant positive effect on civil servants’ mission valence.

In studies examining the relationship between leadership style and civil servants’ public service motivation, servant leadership has been identified as an important promoting factor (Zhang et al., 2024). Servant leadership, by prioritizing the needs of subordinates, empowering employees, building trust, and emphasizing the organization’s ethical and service orientation, aligns closely with the core values inherent in public service motivation, such as altruism and commitment to serving society (Khan et al., 2022). According to Self-Determination Theory, the caring, supportive, and empowering work environment fostered by servant leadership can satisfy employees’ basic psychological needs for autonomy, competence, and relatedness, and the fulfillment of these needs is crucial for eliciting and sustaining public service motivation. When leaders demonstrate exemplary behaviors of serving others and contributing to society, they similarly stimulate employees’ intrinsic public service orientation, thereby enhancing their level of public service motivation (Liu and Zhao, 2022). Based on this, the present study proposes Hypothesis 2b:

*H2b*: Servant leadership has a significant positive effect on public servants’ public service motivation.

Building on the above analysis, servant leadership, through its distinctive behaviors—prioritizing subordinates, empowering employees, and fostering a trusting organizational community—not only directly creates a supportive work environment and enhances employee wellbeing, but also indirectly strengthens public servants’ recognition of and perceived value in the organizational mission (i.e., mission valence) by stimulating their intrinsic public service motivation. In other words, servant leadership may indirectly enhance employees’ mission valence by cultivating their intrinsic public service motivation. Public service motivation may act as a bridging mechanism between servant leadership and mission valence, transmitting the positive influence of servant leadership on mission valence. Based on this, the study proposes Hypothesis 2c:

*H2c*: Public service motivation mediates the effect of servant leadership on public servants’ mission valence.

**Figure 2:**
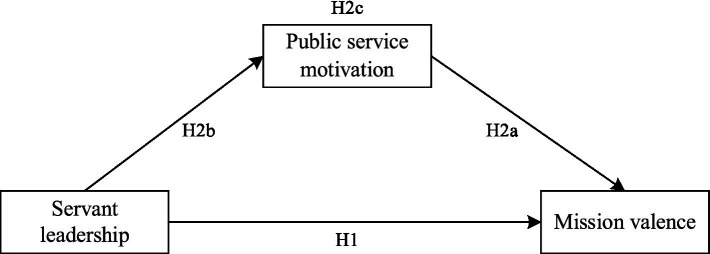
The research model of the study.

Based on the above hypotheses, the theoretical model developed in this study is presented in Figure 2.

### 5.2 Methods

#### 5.2.1 Measurements

This study primarily collected empirical data through a questionnaire survey. The questionnaire consisted of three sections: instructions for completion, respondents’ demographic information, and items measuring the main variables. For the two main variables, Servant Leadership and Public Service Motivation, well-established scales with proven reliability and validity were adopted, while Mission Valence was measured using the scale developed in this study. All three variables were assessed using a five-point Likert scale (1 = “Strongly Disagree,” 5 = “Strongly Agree”).

For Servant Leadership, the study employed the 14-item scale developed by Ehrhart (2004), which has been widely validated. An example item is: “My department manager spends the time to form quality relationships with department employees.” Respondents assessed the behaviors of their direct supervisors.

The measurement of mission valence used the 12-item scale developed in Studies 1 and 2 of this research, encompassing three dimensions: Value Attribute Assessment, Affective Role Identity, and Behavioral Transformation Intention. Given that the Confirmatory Factor Analysis supported the second-order factor structure of mission valence, this indicates that the three sub-dimensions—Value Attribute Assessment, Affective Role Identity, and Behavioral Transformation Intention—share a common higher-order latent variable. Following the recommendations of Chen et al. (2005), given that the second-order model is established, an aggregated score can be used to represent this higher-order construct. Therefore, in the subsequent hypothesis testing, this study treated mission valence as a single, holistic variable. The mean score of all items was adopted as the measurement indicator to examine its influence on the outcome variables.

The measurement of Public Service Motivation used the 10-item scale revised by Coursey and Pandey (2007). An example item is: “Meaningful public service is very important to me.” This scale has demonstrated good reliability and validity.

In addition, this study also collected demographic variables of the respondents, including gender, age, educational level, tenure, department type, and job level, as control variables.

#### 5.2.2 Sample selection and data statistics

This study collected data using a questionnaire survey method, targeting civil servants working in county- and township-level government departments across multiple provinces in China, including Hubei, Henan, Guangdong, Jiangsu, Anhui, Gansu, Sichuan, and Heilongjiang. Given the broad and specific nature of the target population, the study employed a combination of convenience sampling and snowball sampling methods. Data collection was primarily conducted through an online platform, with electronic questionnaires distributed to the target population.

To effectively control for common method bias, this study employed a staggered data collection strategy, with approximately a six-week interval between the two rounds of questionnaire distribution. In the first stage, data were primarily collected on respondents’ demographic characteristics as well as variables such as servant leadership and public service motivation; in the second stage, data on variables including mission valence were collected. By reserving information such as email addresses in the questionnaires for matching purposes, a total of 456 valid questionnaires were successfully matched and screened from the initially distributed surveys, resulting in an effective response rate of 70.15%.

Among all valid respondents, the demographic distribution was as follows: in terms of gender, 48.2% were male and 51.8% were female; regarding age, 84.7% were 35 years old or younger (with 65.4% aged 26–35), indicating a relatively young cohort; in terms of education, 95.6% held a bachelor’s degree or higher (68.4% with a bachelor’s degree, 27.2% with a master’s degree or above); regarding tenure, 75.0% had 6 years of service or less (42.1% with 1–3 years, 25.4% with 4–6 years). The demographic distribution of the sample is reasonably balanced and meets the requirements of the study.

### 5.3 Empirical results analysis

#### 5.3.1 Common method bias and discriminant validity tests

To ensure the quality of the research data, this study first assessed the reliability of the scales used. The reliability analysis indicated that the internal consistency coefficients for the Servant Leadership, Public Service Motivation, and mission valence scales were all above 0.7 (0.958, 0.963, and 0.970, respectively), demonstrating that the scales employed in this study possess good reliability.

Secondly, to address potential common method bias arising from the self-reported nature of the data collection in this survey, this study employed a combination of procedural remedies and statistical tests. In terms of procedural remedies, a two-wave data collection with an interval of approximately 6 weeks was implemented, and anonymity was explicitly assured in the questionnaires to reduce the risk of bias. For statistical assessment, Harman’s single-factor test was employed. An exploratory factor analysis was conducted on all items across the study variables, and the results showed that the first unrotated factor accounted for 48.310% of the variance, which is below the 50% threshold (Podsakoff et al., 2003; Fuller et al., 2016). In addition, the study conducted a supplementary test using the method factor approach to control for unmeasured latent method effects. The results indicated that incorporating the method factor led to only minimal improvements in model fit, with all indices increasing by no more than 0.02 (ΔRMR = 0.11, ΔNFI = 0.009, ΔRFI = 0.004, ΔIFI = 0.007, ΔTLI = 0.005, ΔCFI = 0.007, ΔRMSEA = 0.003) (Liang et al., 2007). Overall, it can be concluded that the data in this study do not exhibit serious common method bias.

Finally, to assess the discriminant validity among the core variables, this study employed Confirmatory Factor Analysis. The results indicate that the dimensional structure of servant leadership demonstrates good discriminant validity (*χ*^2^/df = 4.561; RMSEA = 0.088; NFI = 0.933; IFI = 0.947). Similarly, the dimensional structure of public service motivation also shows good discriminant validity (*χ*^2^/df = 2.084; RMSEA = 0.049; NFI = 0.983; IFI = 0.991), as does the dimensional structure of mission valence (*χ*^2^/df = 2.528; RMSEA = 0.058; NFI = 0.976; IFI = 0.985).

#### 5.3.2 Correlation analysis

This study employed SPSS 27.0 to conduct a correlation analysis of the interrelationships among the three main variables: servant leadership, public service motivation, and mission valence. The specific results are presented in Table 8. The results indicate that servant leadership is significantly positively correlated with mission valence (*r* = 0.446, *p* < 0.001) and with public service motivation (*r* = 0.447, *p* < 0.001). Meanwhile, public service motivation is also significantly positively correlated with mission valence (*r* = 0.665, *p* < 0.01). The above correlation analysis results provide preliminary support for the hypotheses H1, H2a, and H2b proposed in this study, offering initial empirical evidence for subsequent regression analyses and mediation effect tests.

**Table 8:** Means, standard deviations, and correlations among study variables (*N* = 456).

Variables	1	2	3	4	5	6	7	8	9
1. Gender	1								
2. Age	−0.052	1							
3. Education	0.061	−0.057	1						
4. Tenure	−0.054	0.645***	−0.135***	1					
5. Department type	0.049	0.027	0.038	0.006	1				
6. Job level	0.060	0.005	−0.072	−0.020	−0.003	1			
7. SL	0.043	−0.091	0.017	−0.095*	0.035	0.150**	1		
8. MV	−0.081	0.032	−0.070	0.048	−0.007	0.022	0.446***	1	
9. PSM	−0.060	−0.004	−0.067	−0.011	0.064	0.007	0.447***	0.665***	1
Mean	1.520	2.020	2.230	2.800	3.920	1.300	3.725	3.729	3.617
SD	0.500	0.725	0.514	1.131	3.079	0.524	1.007	0.957	0.905

SL, servant leadership; MV, mission valence; PSM, public service motivation. **p < 0.01, ***p < 0.001.

In this context, addressing the high correlation observed between public service motivation and mission valence, this study further conducted theoretical analysis and empirical testing to verify their conceptual independence.

Specifically, from a theoretical perspective, although public service motivation and mission valence are both individual-level psychological variables that involve a focus on public values, there are essential differences between the two in terms of conceptual orientation and psychological attributes:

On the one hand, regarding the target object, PSM is a general and cross-situational service predisposition directed towards abstract public interests or the general public (Perry and Wise, 1990). In contrast, mission valence is a specific, context-dependent psychological assessment directed towards the specific mission of the organization to which the individual currently belongs (Wright, 2007).

Individuals with high PSM aspire to serve society, but this does not imply that they necessarily perceive the specific goals of their current organization as possessing high value. For instance, an employee with high PSM might perceive certain current administrative goals of their unit as cumbersome and meaningless; in this scenario, they possess a high level of PSM but a low level of mission valence.

On the other hand, regarding psychological attributes, PSM is more often viewed as a “Trait-like” personal disposition with relative stability, serving as an antecedent attribute that the individual brings into the organization. Conversely, mission valence is a “State-like” attitudinal perception that changes dynamically under the direct influence of the organizational environment (e.g., leadership style, mission clarity) (Rainey and Steinbauer, 1999).

From a statistical perspective, to rule out the risk of conceptual redundancy, this study conducted a Paired Confirmatory Factor Analysis on the two constructs.

The results indicated that the two-factor model, which treated Public Service Motivation and Mission Valence as two independent factors, demonstrated a good fit (*χ*^2^/df = 1.938, NFI = 0.962, RFI = 0.958, CFI = 0.981, RMSEA = 0.045). In contrast, the single-factor model, which forced the two to merge, showed a significantly deteriorated fit (*χ*^2^/df = 12.795, NFI = 0.746, RFI = 0.720, CFI = 0.761, RMSEA = 0.161).

The Chi-square difference test was significant (△*χ*^2^ = 2271.105, *p* < 0.001). These results suggest that although Public Service Motivation and Mission Valence are correlated, they are empirically distinct constructs.

#### 5.3.3 Regression analysis

This study employed hierarchical regression analysis to examine the relationship between servant leadership and public servants’ mission valence. The results are presented in Table 8. Model 4 indicates that servant leadership has a significant positive effect on mission valence (*β* = 0.520, *p* < 0.001). After controlling for demographic variables, servant leadership accounts for an additional 21.1% of the variance in mission valence, suggesting that higher levels of servant leadership are associated with higher mission valence among public servants. Therefore, Hypothesis H1 is supported. Model 2 shows that servant leadership has a significant positive effect on public service motivation (*β* = 0.488, *p* < 0.001). After controlling for demographic variables, servant leadership explains an additional 20.5% of the variance in public service motivation, indicating that higher levels of servant leadership in the public sector are associated with stronger public service motivation among civil servants. Thus, Hypothesis H2b is supported. Model 5 shows that public service motivation has a significant positive effect on mission valence (*β* = 0.701, *p* < 0.001). After controlling for demographic variables, public service motivation accounts for an additional 43.8% of the variance in mission valence, indicating that the stronger a civil servant’s public service motivation, the higher their mission valence. Therefore, Hypothesis H2a is supported.

#### 5.3.4 Mediation effect test

This study employed the mediation testing method proposed by Baron and Kenny to examine whether public service motivation plays a mediating role in the relationship between servant leadership and mission valence (Baron and Kenny, 1986). As shown in Models 2, 5, and 6 of Table 9, when both servant leadership and public service motivation simultaneously impact mission valence, the regression coefficients of servant leadership (*β* = 0.225, *p* < 0.001) and public service motivation (*β* = 0.605, *p* < 0.001) on mission valence are both significant. However, the coefficient of servant leadership on mission valence decreases from 0.520 to 0.225, indicating that public service motivation partially mediates the effect of servant leadership on mission valence. Thus, Hypothesis H2c is supported. To further rigorously test the mediation effect, this study employed the SPSS 27.0 Process procedure with a bootstrap test, resampling 5,000 times. The results showed that the mediating effect of public service motivation was 0.295, with a 95% confidence interval [0.208, 0.401] that does not include zero. This indicates that the mediation effect of public service motivation is significant, providing further support for Hypothesis H2c.

**Table 9:** Regression analysis results (*N* = 456).

Variables	PSM		MV			
	Model 1	Model 2	Model 3	Model 4	Model 5	Model 6
Gender	−0.117	−0.139	−0.155	−0.179*	−0.073	−0.095
Age	0.005	0.041	0.003	0.041	−0.001	0.016
Education	−0.129	−0.141	−0.116	−0.128	−0.025	−0.043
Tenure	−0.023	−0.003	0.031	0.052	0.047	0.054
Department type	0.022	0.017	0.000	−0.006	−0.016	−0.016
Job level	0.010	−0.116	0.044	−0.090	0.038	−0.019
SL		0.488***		0.520***		0.225***
PSM					0.701***	0.605***
*R* ^2^	0.013	0.218	0.013	0.223	0.450	0.481
Δ*R*^2^	0.013	0.205	0.013	0.211	0.438	0.258
F	0.998	17.873***	0.958	18.396***	52.413***	51.885***

SL, servant leadership; MV, mission valence; PSM, public service motivation; *p < 0.05, ***p < 0.001.

## 6 Conclusion and discussion

### 6.1 Research conclusions and contributions

Rooted in the unique organizational context of China’s public sector, this study focuses on the core cohort of civil servants. Through a series of three qualitative and quantitative studies, we have systematically reconstructed the connotation and structure of civil servants’ mission valence, breaking through the traditional unidimensional concept. We empirically distilled a three-dimensional structure comprising value attribute assessment, affective role identity, and behavioral transformation intention. Furthermore, we successfully developed and validated a multidimensional measurement scale with good reliability and validity. This endeavor aligns with and extends the wider discourse in the world literature of public administration (e.g., Perry, 1996; Rainey and Steinbauer, 1999), which increasingly calls for a more nuanced, multidimensional, and institutionally embedded understanding of public service motivation. Therefore, this work not only provides a precise, localized tool for capturing the intrinsic motivational mechanisms of Chinese civil servants but also effectively expands the theoretical boundaries and explanatory power of mission valence research.

Firstly, Study 1 employed inductive analysis based on grounded theory to advance and deepen the conceptual connotation and structural dimensions of mission valence. Drawing on in-depth interview data from 21 frontline civil servants and utilizing three-level coding analysis, we identified that mission valence is not a singular cognitive state. Instead, it is a complex psychological process encompassing “value attribute assessment” (i.e., cognitive evaluation), “affective role identity” (i.e., emotional connection), and “behavioral transformation intention” (i.e., action orientation). This discovery breaks through the unidimensional perspective prevalent in previous studies, which often focused solely on “job importance” or “emotional arousal” (Wright, 2007; Pandey et al., 2008). It empirically reveals the complete chain within the Chinese public management context—how civil servants’ perception of organizational mission evolves from objective value judgment to internalized emotional identification, and finally converts into behavioral willingness. This multidimensional structural model more comprehensively and profoundly reflects the authentic psychological experience of knowledge-type civil servants, providing a more explanatory and refined analytical framework for subsequent theoretical research.

Secondly, Study 2 strictly adhered to standard scale development procedures to provide the academic community with an effective instrument for measuring the mission valence of civil servants. In this phase, we utilized a sample of 205 for item analysis and exploratory factor analysis, preliminarily forming a measurement tool consisting of 3 dimensions and 12 items. Subsequently, confirmatory factor analysis on an independent sample of 216 confirmed that this three-factor structure possesses excellent model fit, reliability, and discriminant validity. The development of this scale effectively addresses the deficiency of measurement tools in existing research. It not only responds to prior calls for developing mission valence instruments (Yu and Zhang, 2023; Yuan et al., 2024) but also contributes to averting the risk of theoretical stagnation in this field due to a lack of valid measurement. More importantly, this scale is “grown” within China’s unique administrative culture and discourse system. Compared to directly translating Western scales, it more precisely captures the unique psychological characteristics of civil servants under the specific administrative culture and political ethics of the Chinese public sector. Thus, it offers a reliable, localized operational means for future empirical examinations of the formation mechanisms and boundary conditions of mission valence.

Finally, Study 3 preliminarily established the criterion-related validity and theoretical legitimacy of civil servants’ mission valence as an independent construct by introducing external variables. In this phase, we selected servant leadership as the antecedent variable and public service motivation as the mediating variable, conducting correlation and regression analyses based on a sample of 456. The empirical results indicate that servant leadership not only directly and positively influences civil servants’ mission valence but also exerts an indirect effect by stimulating public service motivation. This finding elucidates the formation path of mission valence within the frameworks of conservation of resources theory and the active motivation model. Crucially, it provides preliminary criterion-related validity evidence for the scale. These results demonstrate that the civil servants’ mission valence proposed in this study is neither a simple transplantation of corporate mission sense nor an appendage of other motivational concepts. On the contrary, whether in terms of conceptual connotation, extension, structural content, or essential characteristics, civil servants’ mission valence remains fundamentally distinct from similar concepts such as public service motivation or calling. It stands as an independent construct with unique connotations rooted in the specific context of public organizations, capable of being effectively measured and playing a substantive role within the organizational behavior network. Hence, we believe this validation process attests to the necessity and academic value of developing constructs specifically for civil servants and Chinese cultural contexts of public sectors in behavioral public management research.

### 6.2 Practical implications

This study not only extends the construct of mission valence theoretically but also provides an operational framework for public sector management practice. Based on the research findings, we propose specific managerial interventions spanning measurement tools, organizational policies, leadership development, and individual engagement.

Firstly, the public sector can adopt the civil servant mission valence scale developed in this study as a diagnostic instrument for organizational effectiveness. This tool serves as a quantitative basis for evaluating and optimizing mission education and organizational culture initiatives. Crucially, during pivotal moments such as organizational restructuring, annual performance appraisals, management training, and team building, this instrument enables the quantitative assessment of these activities through pre- and post-testing. By determining whether such initiatives genuinely enhance civil servants’ mission valence, public organizations can timely adjust strategies and optimize resource allocation. The goal is to ensure that mission-related management efforts are internalized by employees, externalized into concrete actions, thereby serving the realization of organizational objectives.

Building on these diagnostic insights, public sector organizations should implement differentiated intervention strategies within human resource policies and job design to address identified weaknesses. For instance, regarding recruitment, the assessment of candidates’ public service values should be prioritized. In terms of induction training, programs focusing on organizational culture—specifically mission and values—should be reinforced to clarify how the organizational mission contributes to national strategies and public wellbeing, thus helping civil servants establish a macro-level perspective. Simultaneously, in the realm of job analysis, it is essential to optimize job design by strengthening the link between daily tasks and the organizational mission. Through mechanisms such as empowerment and authorization, civil servants’ sense of mission can be effectively transformed into proactive engagement and tangible service behaviors. Additionally, public sectors should provide necessary resource support and tolerance for error, encouraging employees to demonstrate initiative in solving public problems and thereby gaining a sense of efficacy.

Moreover, considering the significant role of servant leadership in promoting mission valence, the public sector should place a strong emphasis on cultivating servant leadership and establishing role-modeling mechanisms. By leading by example and acting as practitioners of the mission, leaders can demonstrate a commitment to the public interest that stimulates emotional resonance among subordinates, allowing them to find professional dignity through role identification. To this end, leadership development programs should focus on fostering service awareness, empathy, and empowerment skills among managers. Concurrently, by creating a supportive team climate and attending to the growth needs of grassroots civil servants, organizations can enhance civil servants’ psychological safety and sense of belonging, thereby solidifying the emotional bond between civil servants and the organizational mission.

Ultimately, to ensure that the internalized sense of mission translates into sustained work engagement, it is vital to assist civil servants with their career development planning. By aligning individual career goals with the broader organizational mission, civil servants are encouraged to practice this mission through proactive service behaviors in their daily work.

### 6.3 Limitations and future research directions

This study successfully developed and validated a multidimensional mission valence scale through grounded theory and multi-stage empirical testing. However, several limitations inevitably remain. Acknowledging these constraints is essential for defining the boundaries of our findings and identifying critical avenues for future research.

Firstly, although the construct validity of the scale is established, its conceptual discriminant validity requires further empirical verification. We theoretically distinguished mission valence from organizational identification (Ashforth and Mael, 1989), calling (Dik and Duffy, 2009), and work engagement (Schaufeli et al., 2002). However, these variables were not included in a single model in our study. Future inquiries should measure these constructs simultaneously to confirm the unique explanatory power of mission valence.

Secondly, the cultural specificity of the current scale presents a limitation regarding its generalizability. We employed a bottom-up grounded theory approach to extract items specifically from the Chinese public sector, incorporating unique values such as “family-state sentiment” and the political ethic of “serving the people.” While this context-specific strategy maximized content validity by accurately capturing the psychological characteristics of the target population, it inevitably constrains the scale’s external validity. Consequently, the instrument may not be directly applicable to cross-cultural settings. Subsequent researchers must carefully assess the compatibility of their research context, and priority should be given to cross-cultural validation to test the stability of this three-dimensional structure across different national systems and administrative traditions.

Thirdly, the present study faced significant challenges in obtaining large-scale data due to the closed and specific nature of the Chinese public sector. To complete two independent surveys among grassroots civil servants, we employed a snowball sampling strategy, which addressed certain demographic discrepancies across the different research stages. However, gender, age, educational level, tenure, department type, and job level were included as control variables in our regression analysis, and results indicated that these characteristics did not significantly alter the main relationships. This suggests that the demographic differences did not compromise the robustness of our conclusions. Nevertheless, future research should strive for stratified random sampling to ensure a more balanced distribution of age and rank, allowing for a rigorous examination of the mission valence evolution throughout the civil servant’s career lifecycle.

Fourthly, this study relied primarily on self-reported data, which introduces potential methodological biases. Given the public sector’s culture of political loyalty, respondents may have overstated their alignment with national missions to demonstrate political awareness. Additionally, the exclusive use of positively worded items carries the risk of unconscious acquiescence bias. However, it is worth noting that such responses arguably reflect a genuine internalization of professional norms—a defining psychological characteristic of this group—rather than a mere artifact. To better address these issues, future studies should employ robust designs such as forced-choice or reverse-scored items and utilize triangulation by combining self-reports with supervisor evaluations and objective performance indicators.

Finally, the present study is limited in its ability to draw definitive causal inferences. Although Study 3 utilized data collected at different time points to mitigate cross-sectional bias, this does not constitute a strict longitudinal or experimental design. Consequently, the relationships observed among servant leadership, public service motivation, and mission valence should be interpreted as predictive associations rather than a confirmed causal chain. Future research should employ multi-wave cross-lagged designs to rigorously track the dynamic formation of mission valence over time. Furthermore, subsequent empirical studies should incorporate contextual variables, such as perceived red tape or organizational climate, to comprehensively reveal the boundary conditions and mechanisms at play.

## Data Availability

The original contributions presented in the study are included in the article/supplementary material, further inquiries can be directed to the corresponding author.

## Appendix

**Mission valence scale**

1. The mission of our organization is to provide valuable public services.

2. The mission of our organization is crucial for the revitalization of the nation.

3. The mission of our organization is beneficial to regional social development.

4. The mission of our organization can enhance the wellbeing of the people.

5. The mission of our organization is beneficial to my personal development.

6. I am inspired by the mission of my organization.

7. The mission of our organization to serve the people motivates me.

8. I know the role I can play in carrying out the mission of my organization.

9. The mission of the organization excites me.

10. The mission of our organization fills me with energy.

11. The mission of our organization makes me want to invest myself in my daily work.

12. The mission of our organization makes me eager to serve the people.
