# Correction: Mission valence of civil servants: development and validation of a three-dimensional scale

**DOI:** 10.3389/fpsyg.2026.1787630

**Published:** 2026-02-09

**Authors:** Xi Liu, Mengchu Zhao, Weibo Zheng

**Affiliations:** 1 College of Public Administration, Huazhong University of Science and Technology, Wuhan, China; 2 School of Law and Humanities, Zhejiang Sci-Tech University, Hangzhou, China

**Keywords:** civil servants' mission valence, dimension exploration, grounded theory, public sector, scale development

There was a mistake in Table 8 and Table 9 as published. Specifically, Table 8 and Table 9 currently appear with a yellow background. The corrected Table 8 and Table 9 appear below.

**Table 8:** Means, standard deviations, and correlations among study variables (*N* = 456).

**Variables**	**1**	**2**	**3**	**4**	**5**	**6**	**7**	**8**	**9**
1. Gender	1								
2. Age	−0.052	1							
3. Education	0.061	−0.057	1						
4. Tenure	−0.054	0.645^***^	−0.135^***^	1					
5. Department type	0.049	0.027	0.038	0.006	1				
6. Job level	0.060	0.005	−0.072	−0.020	−0.003	1			
7. SL	0.043	−0.091	0.017	−0.095^*^	0.035	0.150^**^	1		
8. MV	−0.081	0.032	−0.070	0.048	−0.007	0.022	0.446^***^	1	
9. PSM	−0.060	−0.004	−0.067	−0.011	0.064	0.007	0.447^***^	0.665^***^	1
Mean	1.520	2.020	2.230	2.800	3.920	1.300	3.725	3.729	3.617
SD	0.500	0.725	0.514	1.131	3.079	0.524	1.007	0.957	0.905

SL, servant leadership; MV, mission valence; PSM, public service motivation.

^**^p < 0.01, ^***^p < 0.001.

**Table 9:** Regression analysis results (*N* = 456).

**Variables**	**PSM**		**MV**			
**Model 1**	**Model 2**	**Model 3**	**Model 4**	**Model 5**	**Model 6**
Gender	−0.117	−0.139	−0.155	−0.179^*^	−0.073	−0.095
Age	0.005	0.041	0.003	0.041	−0.001	0.016
Education	−0.129	−0.141	−0.116	−0.128	−0.025	−0.043
Tenure	−0.023	−0.003	0.031	0.052	0.047	0.054
Department type	0.022	0.017	0.000	−0.006	−0.016	−0.016
Job level	0.010	−0.116	0.044	−0.090	0.038	−0.019
SL		0.488^***^		0.520^***^		0.225^***^
PSM					0.701^***^	0.605^***^
*R* ^2^	0.013	0.218	0.013	0.223	0.450	0.481
Δ*R*^2^	0.013	0.205	0.013	0.211	0.438	0.258
*F*	0.998	17.873^***^	0.958	18.396^***^	52.413^***^	51.885^***^

SL, servant leadership; MV, mission valence; PSM, public service motivation.

^*^p < 0.05, ^***^p < 0.001.

The original version of this article has been updated.

